# Sub-terahertz feedback interferometry and imaging with emitters in 130 nm BiCMOS technology

**DOI:** 10.1038/s41598-023-43194-8

**Published:** 2023-09-27

**Authors:** Dmytro B. But, Kȩstutis Ikamas, Cezary Kołaciński, Aleksandr V. Chernyadiev, Domantas Vizbaras, Wojciech Knap, Alvydas Lisauskas

**Affiliations:** 1 CENTERA Laboratories, Institute of High Pressure Physics PAS, 01-142 Warsaw, Poland; 2 Institute of Applied Electrodynamics and Telecommunications, Vilnius University, LT-10257 Vilnius, Lithuania; 3 Research Group on Logistics and Defense Technology Management, General Jonas Žemaitis Military Academy of Lithuania, LT-10322 Vilnius, Lithuania; 4 Lukasiewicz Research Network Institute of Microelectronics and Photonics, 02-668 Warsaw, Poland

**Keywords:** Electrical and electronic engineering, Imaging techniques

## Abstract

In this work, we present the effect of self-mixing in compact terahertz emitters implemented in a 130 nm SiGe BiCMOS technology. The devices are based on a differential Colpitts oscillator topology with optimized emission frequency at the fundamental harmonic. The radiation is out-coupled through the substrate side using a hyper-hemispheric silicon lens. The first source is optimized for 200 GHz and radiates up to 0.525 mW of propagating power. The second source emits up to 0.325 mW at 260 GHz. We demonstrate that in these devices, feedback radiation produces the change in bias current, the magnitude of which can reach up to several percent compared to the bias current itself, enabling feedback interferometric measurements. We demonstrate the applicability of feedback interferometry to perform coherent reflection-type raster-scan imaging.

## Introduction

Terahertz (THz) radiation has unique properties, such as penetrating through various dielectric materials or interacting with rotational-vibrational states of many solid compounds, not to mention that it possesses nonionizing photon energy. These and a variety of other characteristics stimulate engineers and researchers to develop and implement novel terahertz emitters and detectors for a broad range of practical solutions such as imaging for biology, medicine^1^, and industry^2^, space research, or high-speed communication.^3^

Silicon complementary metal-oxide-semiconductor (CMOS) technology has taken the lead in digital applications due to its high order of integration, mass production capability, reliability, and well-developed building-block approach. However, typical silicon CMOS technological processes still have a relatively low maximum frequency of oscillation at which the unilateral power gain becomes unity ($f_{\textrm{max}}\approx$ 300 GHz)^4^. Therefore, silicon-based technologies have not been seriously considered for implementing signal sources for the THz frequency range. Instead, materials like InP^5^ and other III-V^6^ group and technologies such as resonant tunneling diodes^7^, Schottky diode-based multipliers^8,9^, quantum-cascade lasers^10^ or difference frequency generation in nonlinear crystals^11^ have taken a lead.

The dominance of III-V high electron mobility devices is gradually decreasing due to the development of bipolar CMOS (BiCMOS) technologies as well as the accessibility of CMOS technologies for a wide range of research groups^12,13^. For example, it is proven that THz detectors fabricated in CMOS allow for an efficient detection at 4 THz and above^14^ as well as for demonstration of a passive detection of human body radiation in THz range below 2 THz at room-temperature conditions^15^.

A number of various experimental approaches enables for the implementation of compact systems for THz imaging with a rapidly evolving new applications^3^. The potential of CMOS structures to operate as compact emitters for the terahertz range was first demonstrated by Huang et al.^16^ & Seok et al.^17^ in 2008. One of the first imaging systems which utilize only CMOS-based sub-THz source and CMOS-based detecor was demonstrated in 2014^18^. It was based on the 220 GHz differential Colpitts oscillator manufactured using a 90 nm Si CMOS process. In 2015^19^, a 160 GHz to 1 THz multicolor imaging system with an antenna-coupled harmonic generator and a multiplier chain implemented in a 250 nm BiCMOS process was presented. The progress in scaling THz sources continues: in 2017, the 500 GHz computed tomography system based on a commercially available 130 nm SiGe BiCMOS technology was created^20^. It used a free-running Colpitts architecture oscillator. A similar CMOS process was used to build a 420 GHz source system-on-a-chip^21^. It was applied for computational imaging with a single-pixel camera and a spatial modulation of the THz radiation. We recently reported a free-space data transmission line^22^, and an imaging system^23^ consisting of an all Si-CMOS THz field-effect-transistor based detector and a voltage-controlled oscillator (VCO). This quasi-optic system allows for achieving incident power-referred signal-to-noise ratio (SNR) of 62 dB in the direct detection regime for one Hz equivalent noise bandwidth at 250 GHz. Such devices can be applied in a variety of compact imaging systems (see a recent review^24^). One of the further possibilities to advance the applicability of electronic-based compact imaging systems is to exploit the reciprocity of electronic components which can be simultaneously used for the emission as well as detection of radiation. Such reflection imaging has been recently demonstrated using a harmonic oscillator implemented in 65 nm CMOS technology^25^.

Here, we present a comprehensive study of the dynamical properties of fundamental frequency electronic oscillators subjected to feedback radiation and its application for reflection-type coherent imaging^26^. The principle of feedback interferometry is well known in the optoelectronic community; however, until now, it has been employed mainly in visible and near-infrared optics domains^27,28^. In the THz frequency range, the quantum cascade laser (QCL) was successfully applied for the construction of laser self-mixing interferometry^29–32^. Furthermore, the successive exploitation of feedback-induced signal has been recently predicted and reported using a resonant tunneling diode transceiver in the 300 GHz frequency band^33,34^. The aforementioned imaging application with a CMOS-based harmonic oscillator went a step further and proposed an interpretation that the data contains spectroscopic information up to 1.4 THz^25^ which can be even extended for over 2 THz. However, the phenomenon of feedback-induced effect on oscillator operation conditions has not been accounted. When the system becomes nonlinear, i.e., the feedback alters the operation point of the oscillator, the standard Fourier-transform method does not represent true harmonic content. To the best of our knowledge, our contribution is the first to utilize a feedback interferometry phenomenon for THz imaging with an electronic circuit-based fundamental frequency oscillator, also enabling us to clarify the issue of feedback-induced spurious harmonic content.

## Experimental setup

**Figure 1:**
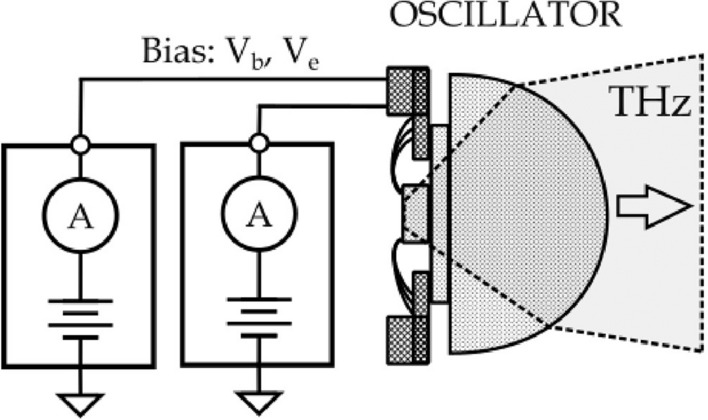
Schematic representation of the oscillator assemblies. The construction: a hyper-hemispheric high resistivity silicon lens with a 12 mm diameter and an undoped high resistivity silicon carrier wafer glued to the printed circuit board with bonding pads and protection electronics.

**Figure 2:**
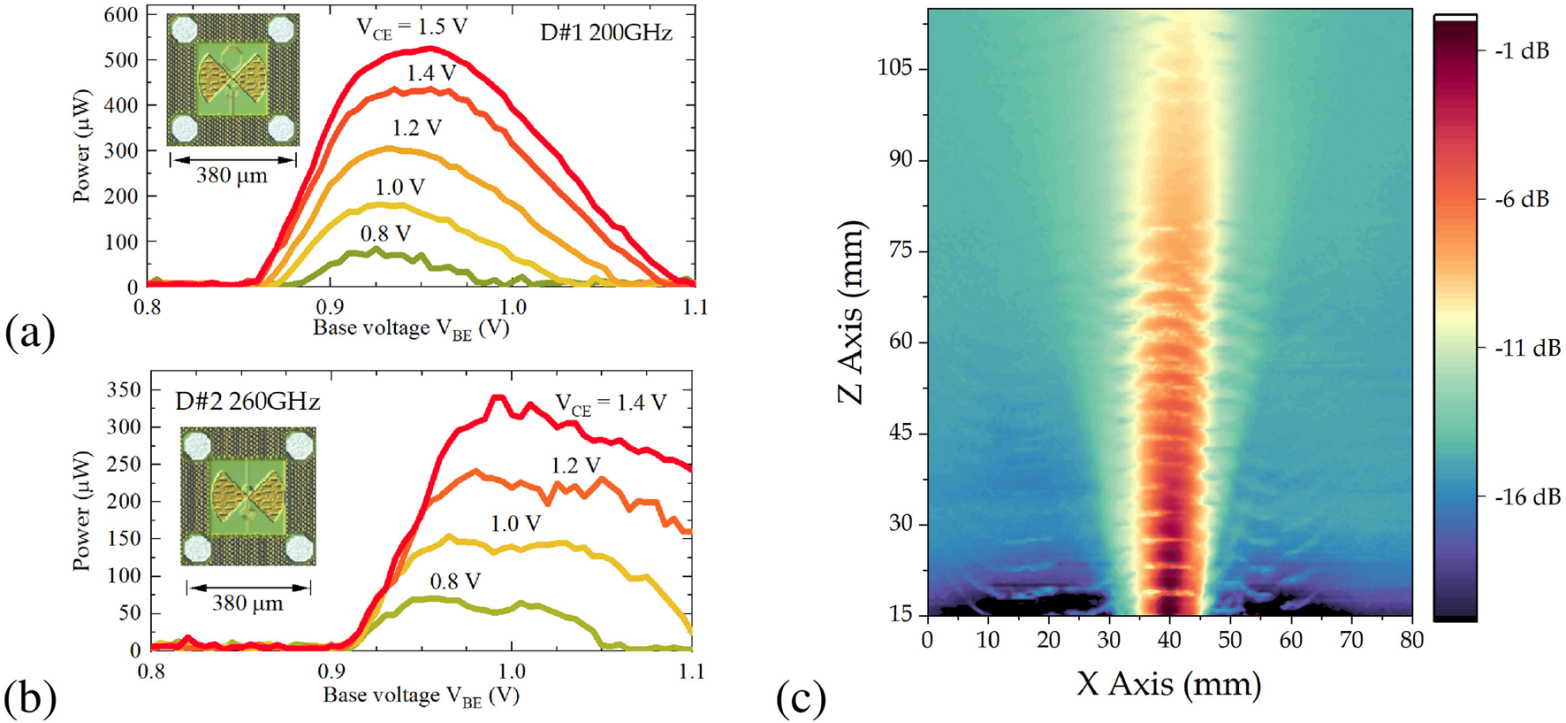
Total radiation power as a function of V$_\text {BE}$ bias at different collector-emitter biases V$_\text {CE}$ for two voltage-controlled oscillators: D#1 200 GHz (**a**) and D#2 260 GHz (**b**). The inset shows a microphoto of integrated VCO with a substrate antenna and contact pads. (**c**) The beam profile of D#2 260 GHz in the X and Z directions, measured with a resonant TeraFET detector^35^.

For this work, we implemented two fundamental frequency VCOs in SiGe 130 nm BiCMOS SG132G technology provided by the IHP GmbH, Frankfurt (Oder) foundry. These sources are based on the Colpitts oscillator concept (see Supplementary S.1). Detailed information on used electronic elements is given in Supplementary S.2. Our design is optimized for the first-harmonic emission into the free space through the substrate side with a high-resistivity silicon hyperhemispheric substrate lens as presented in Fig. 1. The lens has a diameter of 12 mm with a height of 6.8 mm. Devices are glued onto a 525 $\mu$m-thick high-resistivity silicon carrier. The scanned emission profile of the second device (abbreviated as D#2 260 GHz) in the X (lateral) and Z (vertical) directions, measured with a resonant TeraFET detector, where the Y direction is parallel to the E-field of copolarized detector and emitter, is presented in Fig. 2c. This figure shows the distribution of radiated power measured with a small effective area detector (FET coupled to the patch antenna), i.e., it is proportional to the intensity distribution. The analysis of the measurements after transformation to spherical coordinates allows us to estimate a half-power beamwidth of about 15 degrees with a resulting maximum directivity of 23.7 dBi or over 20 dBm EIRP for our source.

**Figure 3:**
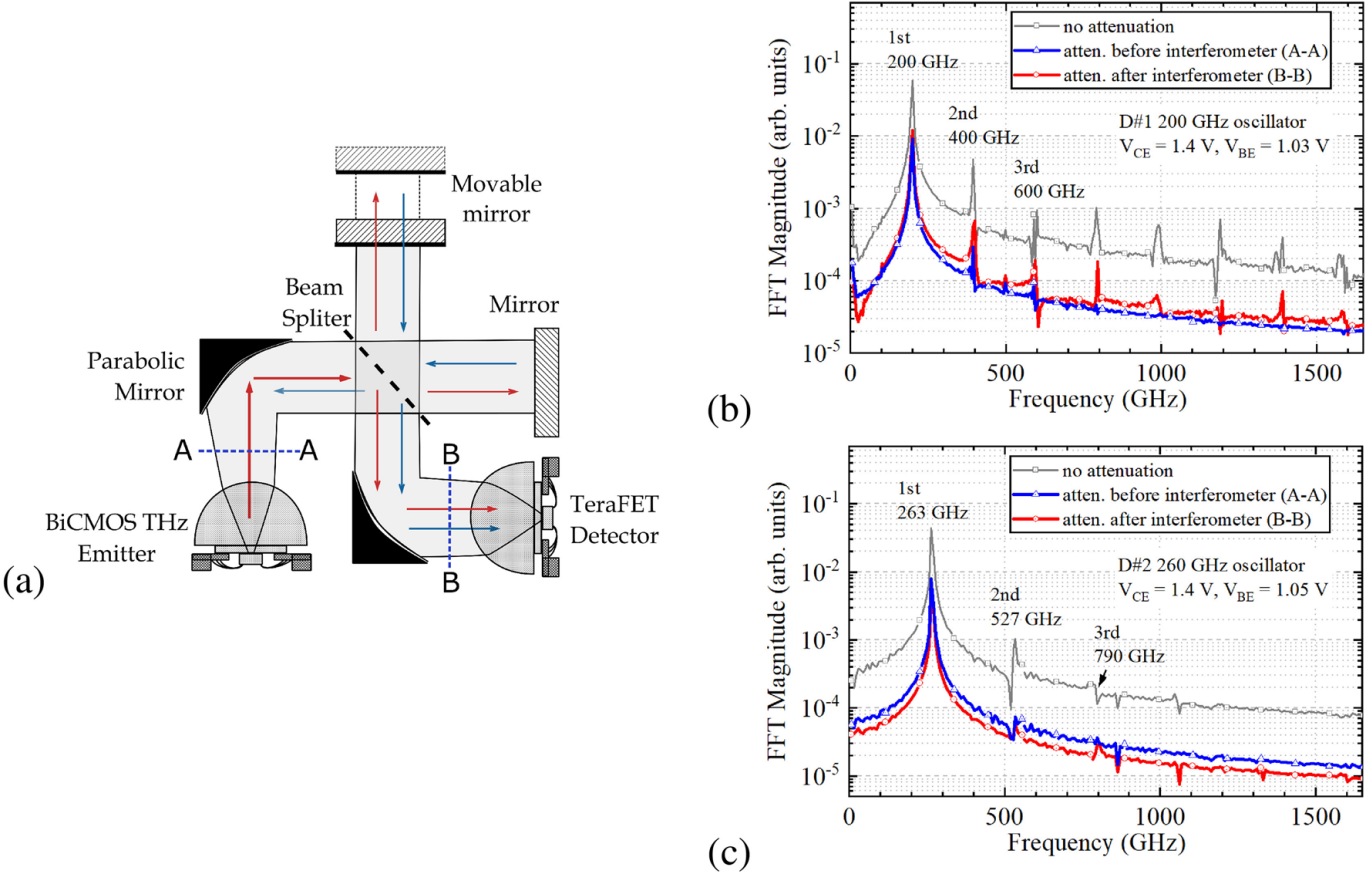
Radiation spectrum obtained using Fourier transform interferometry techniques (**a**) for two 130 nm SiGe HBT fundamental oscillators: D#1 200 GHz (**b**) and D#2 260 GHz (**c**). The spectra were recorded using an 11 dB attenuator at two positions: before (marked as A–A, blue triangles) and after (B–B, red circles) the interferometer. Also, one spectrum was recorded using no attenuator (grey squares).

The emission characteristics as a function of base and collector bias voltages are shown in Fig. 2. Figure 2a presents results for device D#1 (200 GHz design, shown by the inset microphoto) reporting radiated power exceeding 500 $\mu$W—which constitute only about 25% of the value predicted by our simulations. The maximum power obtained for the device D#2 (see Fig. 2b) reaches 325 $\mu$W achieving less than 12% of the value predicted by simulations (see Supplementary Fig. S3). Whereas a loss for the D#1 can be attributed to a combination of 30% Fresnel loss at the silicon-air interface, a 30% loss in 280 $\mu$m-thick p-doped substrate with 10 $\Omega$cm resistivity and about 50% by antenna efficiency, the additional 50% loss for device D#2 currently remains unaccounted for. The nearly 100 mV shift in base bias between the simulated and experimentally measured characteristics can be attributed to a voltage drop on about 3 $\Omega$ additional resistance introduced by the analog switch in biasing circuitry. Contrasting to simulated on-chip 8% DC-to-RF conversion efficiency, the measured radiated efficiency values stay somewhat lower: 1.3% for D#1 and 1.2% for D#2 designs.

For a coarse determination of the oscillation frequency, we have employed a home-built Michelson interferometer combined with a wideband TeraFET THz detector^15^. Figure 3a presents the implemented setup. Spectra obtained for both HBT-based fundamental oscillator sources exhibit the radiation peaks with the highest power at the fundamental frequency of 200 GHz (D#1, Fig. 3b) or 260 GHz (D#2, Fig. 3c). A higher harmonic content showing spectral peaks even above 1.5 THz (see Fig. 3b) raises serious doubts about their validity. According to the definition, the Fourier transform of the autocorrelation signal corresponds to the radiation spectrum only if the systems are time-invariant and linear. However, if a feedback from the measurement system alters the operation point, this analysis can bring spurious content. To determine, which of the higher harmonic lines are real and which are spurious, we performed three measurements: without any attenuation (gray squares), with an 11 dB attenuator placed before the interferometer in the plane A-A (the blue line with triangles shows results), and the same attenuator placed after the interferometer in the plane B-B, just in front of the detector (the red line with circles shows data). Two last experiments allow us to maintain the same power level on the detector while getting a different level of the signal reflected from fixed and moving mirrors back into the source, indicated by the blue arrows in Fig. 3a. Finally, by comparing the difference in calculated spectra (lines with red circles vs. blue triangles), one can conclude that most of the spectral lines at higher harmonics originated from the feedback.

To cross-check the results of frequency determination, we utilized a measurement setup to determine the oscillator frequency precisely. This was achieved using heterodyne mixing between the test emitter and the reference generator. The radiation emitted by the emitter was overlapped using a beam-splitter and the tunable source based on the Schottky diode frequency multiplier chain (Virginia Diodes, Inc.). The signal’s linewidth is determined by the modulation bandwidth of the detector, which is currently limited to 4 MHz.

## Opto-electronic feedback

Most electronic devices, including the fundamental VCO, are prone to radiation self-mixing effects resulting from the feedback. Although this feedback, in most cases, is undesired, such a feedback-originating signal could offer a practical use as an additional source of information in a detection system. Self-mixing interferometry, also known as feedback interferometry, is based on the mixing of the radiation reflected from external target with the electrical oscillations internally produced by the emitter. In other words, the radiation stemming from the one reflected by an obstacle or the object radiation can get mixed with an intrinsic electric field and, concomitantly, can be traced by monitoring the electrical operation parameters of the source. The best-known systems with electronic-optical feedback are lasers, in which part of the emitted light is sent back to the internal cavity, allowing changes to occur in the corresponding temporal waveform. For example, such feedback effect in the THz range was reported with radiation derived from mid-infrared lasers^36^ or directly with quantum cascade lasers^37^, and resonant-tunneling diodes^38^.

**Figure 4:**
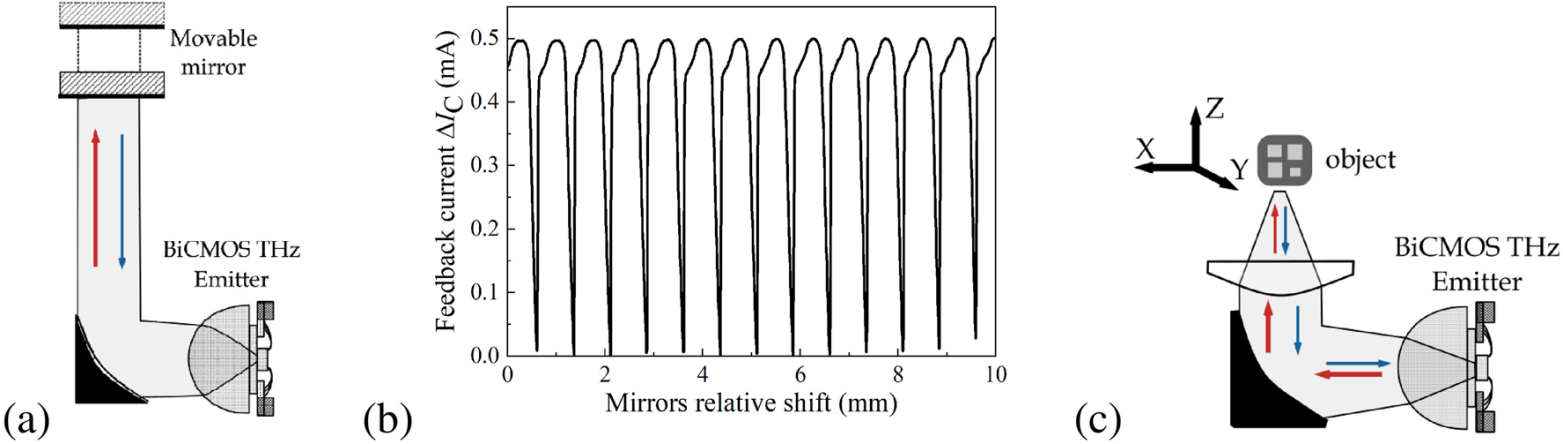
(**a**) The schematic of the feedback effect study setup. (**b**) Fluctuation in collector-emitter current caused by the self-mixing effect caused by the oscillator radiation reflected from the flat mirror. The fluctuation amplitude could achieve 0.5 mA at 17 mA (D#2) of consumable current. (**c**) Schematic of the experimental setup for imaging using the feedback effect. An object is placed on XYZ motorized stage.

The self-mixing in the electronic source can be investigated by reflecting the collinear beam with a flat metallic mirror, as shown in Fig. 4a. Such feedback alters the bias current, whose periodic dependence on the relative mirror shift is shown in Fig. 4b, proving the coherent nature of the signal. The example shows the amplitude of device D#2 collector current oscillation at V$_{CE}$ = 1.3 V and V$_{BE}$ = 1 V. The $\Delta I_C$ is obtained after subtracting about 17 mA DC component of the collector current. The amplitude of the self-mixing signal reaches about 3% of the DC current. This signal shows a strong deviation from a usually expected sinusoidal shape. It originates from the feedback radiation-induced effect on oscillator performance. Once the feedback is reduced, the shape resembles the sinusoidal form and has been confirmed in the previous discussion of the results presented in Fig.3

**Figure 5:**
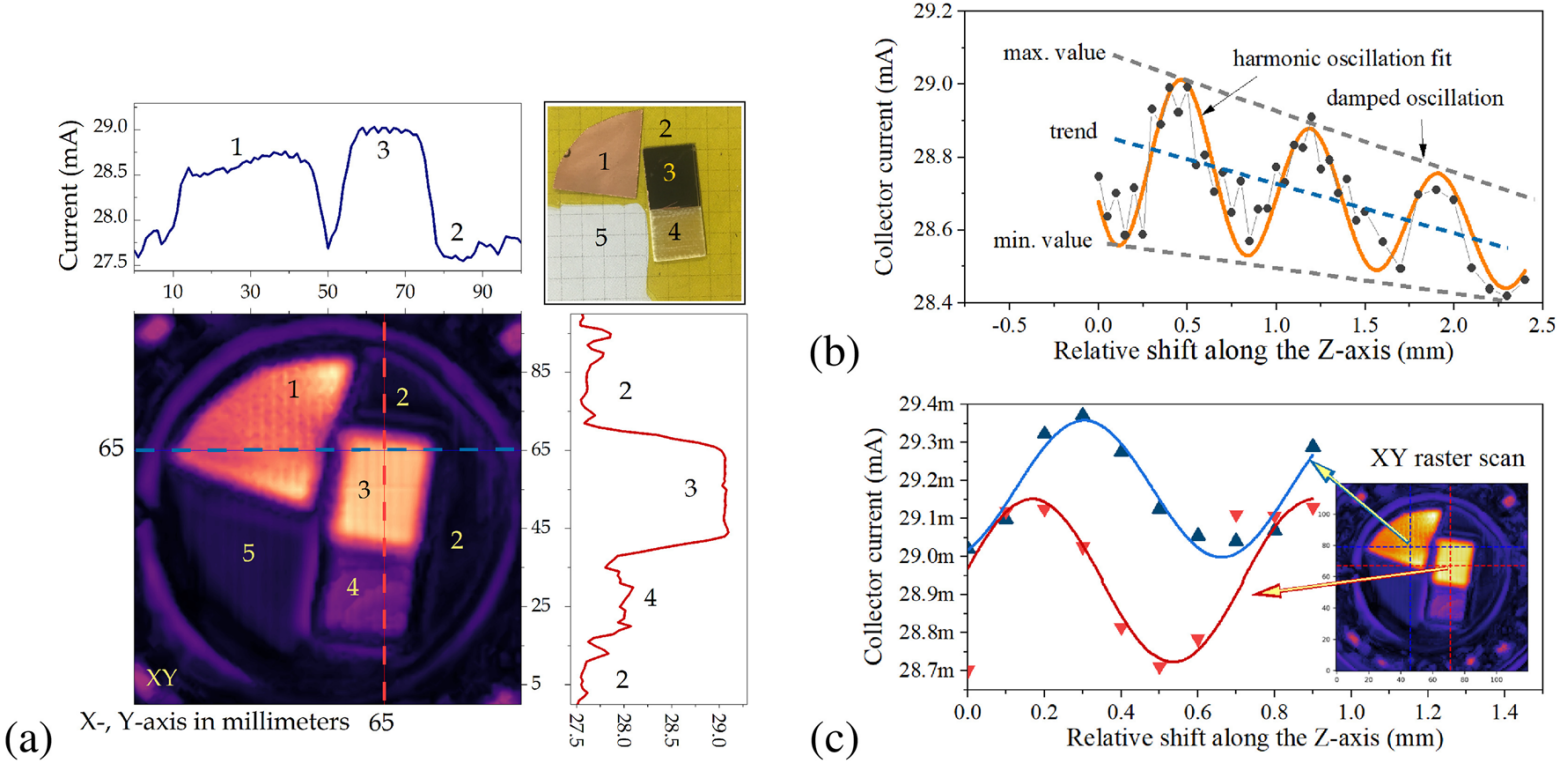
(**a**) Raster scan obtained via D#1 collector current sensing with feedback influence. The step size is 0.5 mm. The top and right panes show collector current levels at positions marked by blue and red lines, respectively. The top right insert shows the photo of the object: 1 is the cooper foil; 2 is the $7\,\upmu \hbox {m}$ Mylar foil (background - substrate), 3 is the FR4 laminate with cooper, and 4 is the FR4 without cooper; 5 is empty space. (**b**) Typical dependency of collector current from a relative shift in the Z direction. The grey lines mark extreme values of current oscillations; the blue line is the trend, and the orange is the harmonic oscillation fit to experimental data. (**c**) Comparison of collector current as a function of a relative shift in the Z-direction at two different positions.

A change in the bias current produced by the self-mixing effect can be measured either in the dc regime or by employing signal modulation techniques and used to form reflection-type images. This also allows for the simultaneous use of the emitter as a sensitive detector. Figure 4c shows a schematic of the experimental setup for imaging. The setup consists of the emitter on the hemispherical 12 mm lens, the 2” parabolic mirror (PM) with parent focal length 2”, and plano-convex aspheric TPX (poly 4 methyl pentene-1) lens with Ø1.5” and effective focal length 2”. An object is mounted on the XYZ motorized stage, where the Z-axis is in line with the optical axis. The setup acquires images by raster-scanning the sample through the focused THz beam while recording the actual collector current value on a ’pixel-by-pixel’ basis. Figure 5a shows a 2D map of the collector current change in the XY-plane. The test object is shown in the top right corner of the figure and consists of the cooper foil (1), the substrate $7\,\upmu \hbox {m}$ Mylar foil (2), the FR4 laminate with cooper (3), the FR4 without cooper (4), and the space of scan without any object (5).

Typical dependence of a collector current as a function of a relative object shift through the focus spot in the Z-direction is shown in Fig. 5b. When moving away from the lens focal point, the signals present damped harmonic oscillation. The signal shows a trend-line behavior (the blue dashed line) that we suppose is correlating with the nonlinear process of self-mixing. It can be noted that the maximal amplitude change in the collector’s current reaching up to 0.5 mA well correspond to the change registered in a planar configuration (see Fig. 4a). The simulated low-frequency current noise of the transistor has two contributions: 1/*f*-type noise at low frequencies and ”white”-type noise with a density of current fluctuations of 170 pA/$\sqrt{\textrm{Hz}}$ above the 1/*f* noise corner frequency at about 6 kHz. This allows us to determine the ultimate limit for the sensitivity of the homodyne receiver to the change in the power to reach $5\cdot 10^{-4}\,\textrm{W}\cdot \left( 170\cdot 10^{-12}\,\mathrm {A/\sqrt{Hz}}/5\cdot 10^{-4}\,\textrm{A} \right) ^2 \approx 5.8\cdot 10^{-17}$ W/Hz. Although the noise in the currently acquired data is dominated by read-out electronics, we are certain that the sensitivity of the optimized system might strongly outperform a few fW/Hz sensitivities reported for patch-antenna coupled FET-based heterodyne sensors^39^.

**Figure 6:**
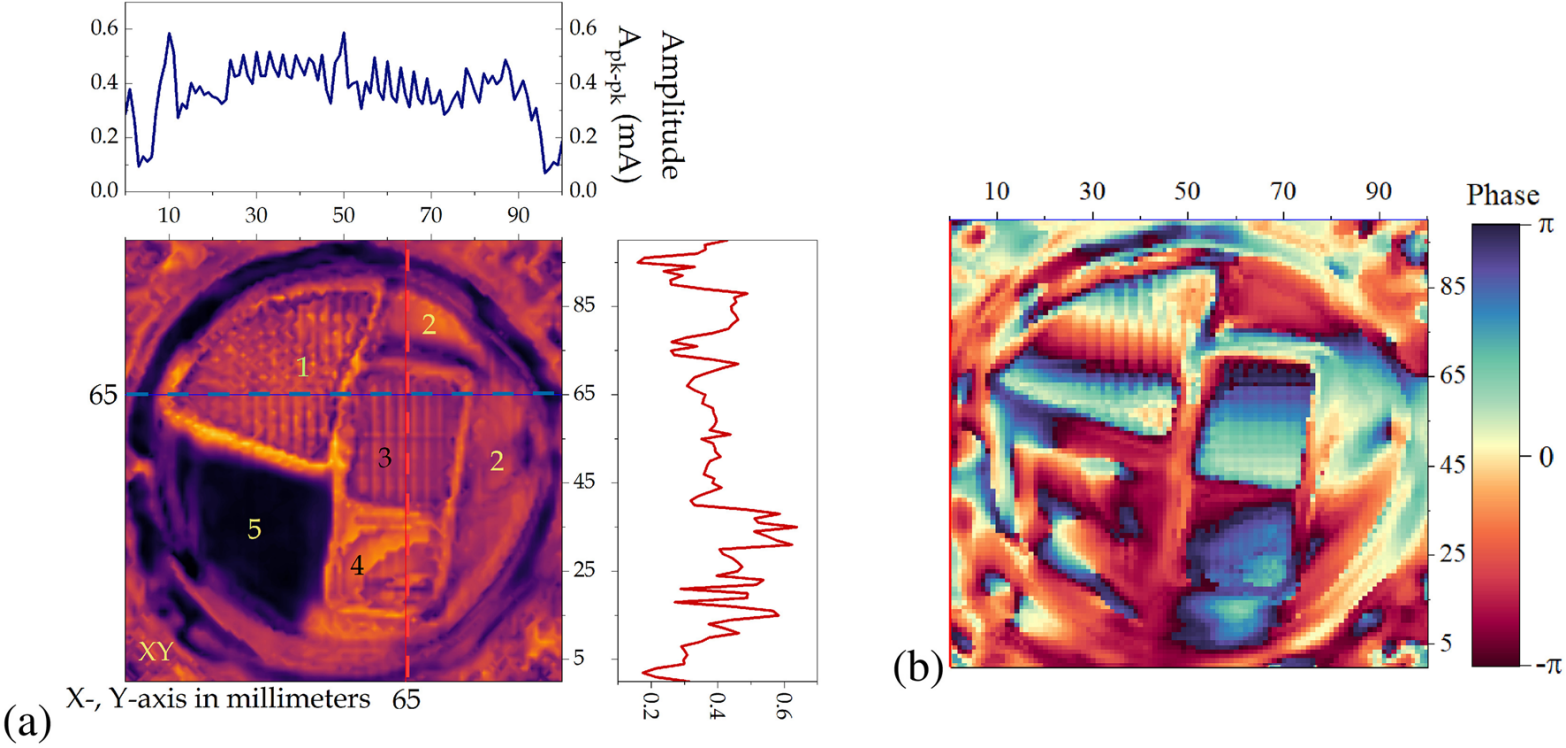
(**a**) 2D image of feedback amplitude for Fig. 5a data. The top and right panes show amplitude feedback current oscillation at positions marked by blue and red lines. (**b**) Image of the relative phase of the feedback oscillation.

Figure 5c presents the values for collector current in the dependency of a relative shift in the Z-direction at two distinct positions (the data in Fig. 5a). For a reconstruction of a feedback image, we analyze oscillation amplitude and phase in each point of the raster scan. A shift of the object within 1.3 mm distance is enough to cover 1.5 of the oscillation period. An oscillation trend is subtracted from each curve. Finally, we determine the peak-to-peak amplitude in each point, which is presented in Fig. 6a. The following step analyzes the phase shift concerning reflection from the metal surface (3). The phase shift was evaluated using a relatively simple routine. A sinusoidal form was created and compared with the measured amplitude as a minimal relationship between the two data vectors. Data fitting was done using a double ”for” cycle by creating multiple Z-shift values (1/z from 0 to 1/z step) and phase shift (from $-\pi$ to $\pi$) combinations for the sinusoidal curve and measured data correlations. The best fitting 1/z ”frequency” for free space (that had the most cases of the highest correlation coefficients) was used on the whole image, and the result is shown in Fig. 6b.

As a summary, we can use the total intensity of the reflected signal (as example Fig. 5a) as an additional source information for transmission imaging. This data allows to separate absorbing regions from the ones with metal-like reflection. The amplitude of the reflected signal could provide information about the borders of objects. As it is shown in Fig. 6a, even Mylar film (4) with low reflectivity can be discriminated from empty space (5). Figure 6b indicates that there is a tilt between the incident and reflected beams and the fact that the metallic foil surface (1) is strongly bent in comparison to the metal on top of FR4 (3). The phase image also carries information about the changes in optical thickness or heights; however, a more sophisticated reconstruction algorithm must be employed.

Furthermore, the application of feedback interferometry based on terahertz transistor oscillators is not limited to the demonstrated raster-scan imaging scenario. It can be employed in a wider field of applications, such as near-field imaging with sub-wavelength resolution, for tracing chemical absorption lines in the THz frequency range or precise distance sensors.

## Conclusion

In this work, we have demonstrated the feedback self-mixing imaging system based on a 200 GHz and 260 GHz BiCMOS VCO operating as a single-pixel coherent emitter-detector. Devices produce 525 $\mu$W and 325 $\mu$W emissions with 1.3% and 1.2% DC-to-RF power conversion efficiency. We demonstrate that a feedback into the source results in self-mixing and can be monitored via the change in DC bias current effect. We applied this phenomenon by monitoring the phase and amplitude of formed reflection-type images. Besides the demonstrated application to imaging, feedback interferometry with compact emitters can be useful for a wider field of applications, such as tracing chemical absorption lines or measuring distance accurately in the THz frequency range.

### Supplementary Information

Supplementary Information.

## Data Availability

The datasets used and/or analysed during the current study available from the corresponding author on reasonable request.
