# Supplementary material for: Sub-terahertz feedback interferometry and imaging with emitters in 130 nm BiCMOS technology
Source: Sci Rep. 2023 Sep 27;13:16161. doi: 10.1038/s41598-023-43194-8 (PMC10533495; doi:10.1038/s41598-023-43194-8)
Supplement: Supplementary file 1 — Supplementary Information. [file 41598_2023_43194_MOESM1_ESM.pdf]

# Supplementary information
## Sub-terahertz feedback interferometry and imaging with emitters in 130 nm BiCMOS technology

**Dmytro B. But [1,*], Kęstutis Ikamas [2,3], Cezary Kołaciński [1,4], Aleksandr V. Chernyadiev [1,*], Domantas Vizbaras [2,*], Wojciech Knap [1,*], and Alvydas Lisauskas [1,2,+]**

[1]CENTERA Laboratories, Institute of High Pressure Physics PAS, Warsaw, 01-142, Poland
[2]Institute of Applied Electrodynamics and Telecommunications, Vilnius University, Vilnius, LT-10257, Lithuania
[3]Research Group on Logistics and Defense Technology Management, General Jonas Žemaitis Military Academy of Lithuania, Vilnius, LT-10322, Lithuania
[4]Lukasiewicz Research Network Institute of Microelectronics and Photonics, Warsaw, 02-668, Poland

## Device Structure, Oscillation Principle, and Oscillation Characteristics

### S.1 Colpitts Oscillator basics

There are different methods for generating THz radiation using electronic concepts. For example, the electrical nonlinearity of the electronic component can be exploited for frequency multiplication, or the amplifier with positive feedback can be used to create the oscillator. Depending on the nature of amplification and the peculiarities of implementation, exist various topographies of oscillators.

A typical schematic of the Colpitts oscillator in a common-collector configuration is presented in Fig.S1(a) [1]. The general functionality of an oscillator requires only a single amplifying element (it can be either a bipolar transistor or a field-effect transistor), one inductor $L_B$, and two capacitances $C_1$ and $C_2$. The oscillation frequency is defined by the resonant circuit formed by the inductor and the capacitance value resulting from the in-series connection of capacitances $C_1$ and $C_2$:

$$f_{res} = (2\pi\sqrt{(L_B C_1 C_2/(C_1 + C_2))})^{-1} \qquad (1)$$

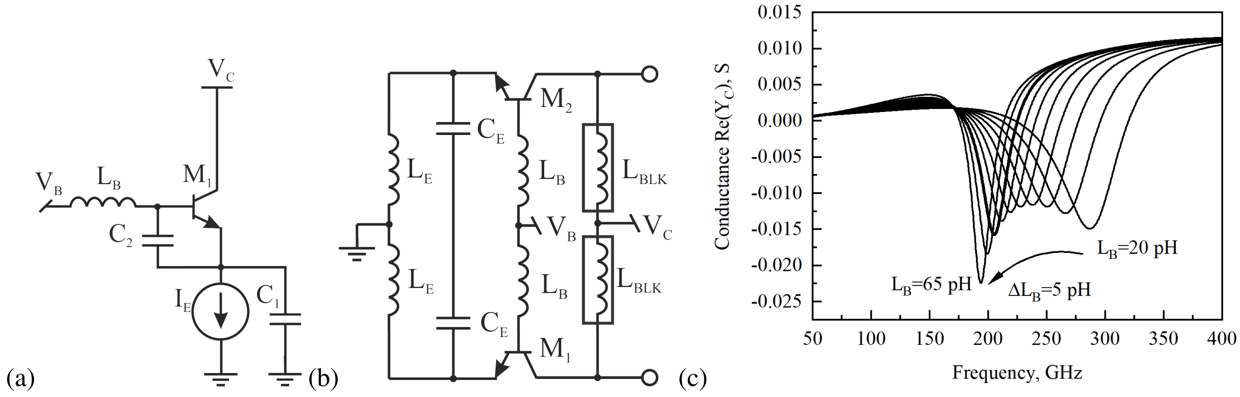


**Figure S1.** a) A simplified schematic of the common-collector Colpitts oscillator. b) A differential Colpitts-type topology with a pair of bipolar transistors $M_1$ and $M_2$, which can be interpreted as a one-port element with negative conductance. The elements $C_1$ are substituted with $C_E$. To achieve the highest frequency operation, the capacitor $C_2$ can be omitted as its role can be taken by internal capacitance $C_{BE}$. c) Simulated real part of complex conductance of the circuit shown in part (b). The value of $C_E$ is 12 fF, the inductance $L_E$ - 90 pH. Transistors $M_1$ and $M_2$ are identical 4-finger devices from SG13G2 process. The simulation shows that resonant frequency with approximately similar minimal value of negative conductivity can be effectively tuned from below 200 GHz to nearly 300 GHz by reducing the value of $L_B$ from 65 pH to 20 pH.

One of the critical aspects of the simple circuit, as presented in Fig. S1(a), is a requirement for a voltage gain to exceed 4 to sustain stable oscillations. However, this requirement can be eased by implementing a differential topology with two transistors $M_1$ and $M_2$ as presented in Fig. S1(b).

The differential circuit implementation has several advantages. First, it allows to sum on the power of two identical oscillators or extend the bandwidth by the asymmetry of sub-circuits as they can operate independently. Second, it provides a virtual ground along the symmetry axis, which can be used to bias the oscillators[2]. Furthermore, the oscillator analysis can be simplified into a one-port device treatment. The illustrative simulation of the real part of complex conductance between collector terminals is shown in Fig. S1(c). In simulations, the value of $C_E$ was fixed to 12 fF, and the inductance $L_E$ - 90 pH. Transistors $M_1$ and $M_2$ are two identical 4-finger devices from SG130G2 process. The value of $C_{BE}$ can be approximately estimated from technology typical 2.5 fF/$\mu$m[3]. The simulation shows that resonant frequency with the minimum negative conductivity can be effectively tuned from below 200 GHz to nearly 300 GHz by reducing the value of $L_B$ from 65 pH to 20 pH. Furthermore, the modelling indicate that one can directly use a resistive load between collector terminals to extract the radiation.

## S.2 Oscillator implementation

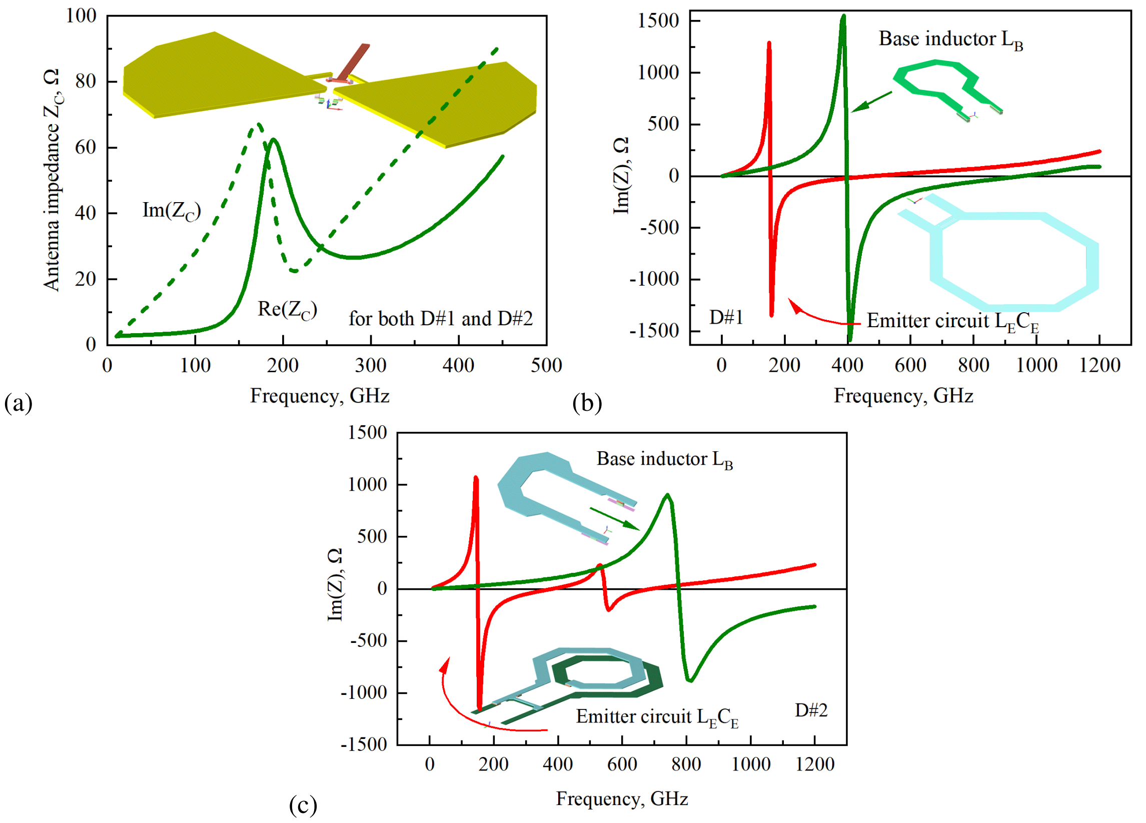


**Figure S2.** a) Real and imaginary parts of simulated antenna impedance. The inset presents a 3D sketch of a modified bow-tie antenna used for both oscillators. b) Spectrum of the imaginary part of impedance for gate's inductive element and source's LC circuit for D#1 200 GHz device. c) Spectrum of the imaginary part of impedance for gate's inductive element and source's LC circuit for D#2 260 GHz device.

In order to implement the oscillators, one has to design the main passive elements (capacitors and inductors) as well as optimize their interplay with transistors. We have selected to use 4-finger devices from IHP SG13G2 technology with $f_T$ and $f_{\max}$ of 350 GHz/450 GHz. Fig. S2(a) presents the real and imaginary parts of simulated antenna impedance to be used to load the oscillator. It has a bow-tie form with the shorting element as presented by a 3D sketch shown in the inset of Fig. S2(a). The metallic connection of leaves of bow-tie lowers the real part of the impedance to 25-40 Ω above 200 GHz with about equal inductive contribution. The same antenna was used in both devices: D#1, which targeted fundamental oscillation frequency at 200 GHz, and D#2 at 260 GHz. The simulated spectrum of main reactive elements and their forms are presented in Fig. S2(b) and (c). We have designed two different implementations of circuits for emitter $L_EC_E$ with comparable values of equivalent

inductance and capacitance. The main element which defined operating frequency was base inductance $L_B$. The resulting parameters are listed in Table S1.

**Table S1.** The parameters of simulated VCO elements.

| | D#1 200 GHz | | D#2 250 GHz | |
|---|---|---|---|---|
| Circuit Element | Lumped value | Impedance | Lumped value | Impedance |
| Load ($2Z_C$) | 21.7 pH | 34.4+i27 | 15 pH | 23.4+i24.3 |
| Emmiter C ($C_E/2$) | 5.3fF | 24.7-i141.4 | 6.3 fF | 11.7-i100.7 |
| Base inductor ($2L_B$) | 118 pH | 9.9+i148.7 | 37.7pH | 5.8+i59 |

The simulated on-chip performance of both devices is presented in Fig. S3 panels (a) and (b). Simulations predict peak on-chip power of more than 2 mW with almost no frequency tunability by base bias voltage for D#1 and about 6 GHz tuning range for D#2. We would like to point-out that the Eq. 1 and simulated parameters given in the Table S1 with the value $C_{BE} \approx 9$ fF doesn't allow to predict resonant frequency as presented in simulation results in Figs. S1(c), S3(a) and S3(b) indicating that analytic treatment must include other elements of high-frequency equivalent circuit.

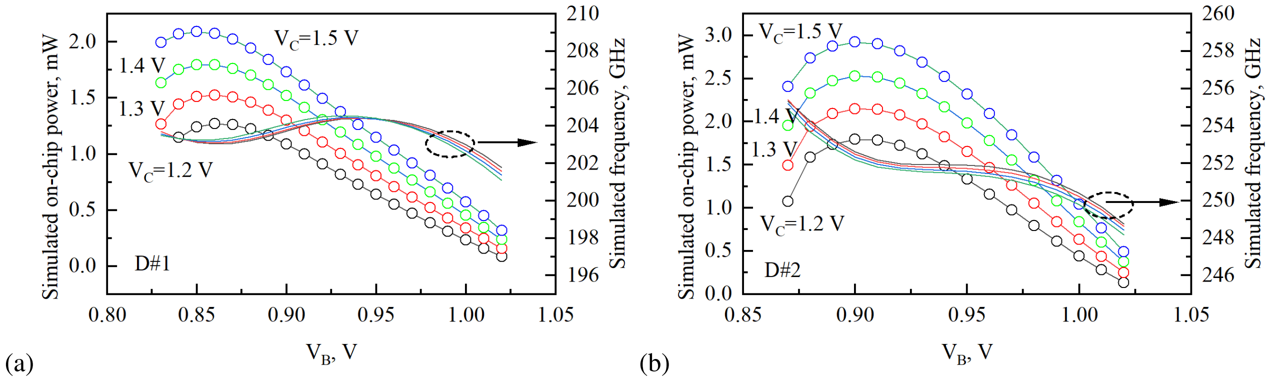


**Figure S3.** Simulated power (symbols, left axis) and frequency (right axis, lines) for (a) device D#1 with a target frequency of 200 GHz and (b) device D#2 with a target frequency of 260 GHz.

## Supplementary References


1. Kęstutis Ikamas, Dmytro B. But, Albert Cesiul, Cezary Kołaciński, Tautvydas Lisauskas, Wojciech Knap, and Alvydas Lisauskas. All-Electronic Emitter-Detector Pairs for 250 GHz in Silicon. *Sensors 2021, Vol. 21, Page 5795*, 21(17):5795, aug 2021.
2. Bassam Khamaisi and Eran Socher. A 209-233 GHz frequency source in 90 nm CMOS technology. *IEEE Microwave and Wireless Components Letters*, 22(5):260–262, 2012.
3. Niccolò Rinaldi and Michael Schröter. *Silicon-Germanium Heterojunction Bipolar Transistors for Mm-wave Systems Technology, Modeling and Circuit Applications*. River Publishers, New York, 1 edition, September 2022.
